# Management of Albuminuria

**Published:** 1876-08

**Authors:** 


### The Management of Albuminuria.

In an article in the London *Medical Times and Gazette,* Dr. W. H. Dickinson, of London, writes:—

To give rest, as far as may be, to an inflamed structure, is an old sound maxim ; and it is not less obvious, in regard to the system at large, that if a great channel of exit be obstructed, the materials which therefore tend to accumulate should be sparingly introduced. The diet with albuminuria, save with that of lardaceous origin, in which the secreting power is until late little interfered with, while an exhausting discharge may have to be obviated, should be below the custom of health in its nitrogenous components. It may abound in milk and farinaceous matter, while fish may often take the place of flesh. The increase of albumen in the urine, upon a too early resort to a meat diet, is a common experience. With regard to liquids, it cannot be too strongly insisted upon that the functional strain upon the kidney is not to be measured by the quantity of water which filters through it, but by the quantity of refuse, mainly nitrogenous, which it has to convert and eliminate. Water, which probably transudes almost as through dead membranes, probably makes little demand upon the real secretive function. The worst kidneys, indeed those most hopelessly incapable of their special work, will often discharge most of it; and it is easy to see that its passage, not to be regarded as the result of glandular effort, is salutary, both in the dilution of scanty and irritating urine, and also in washing out the solid products which, under the inflammatory process, collect mischievously in the tubes. A further use is to be discerned in this law. The solids of the urine vary with its water. With given kidneys, the solid excreta wax and wane with the bulk of the urine. Any means, therefore—mere aqueous filtration as safely as any—which increase this will also magnify the components of the secretions which are essential to life. With tubal nephritis, therefore, and scanty urine, an aqueous dietary, even with the addition of distilled water, or the element in some slightly sophisticated shape, will prove in every sense beneficial. In many, perhaps in most, cases of nephritis of tabul origin these remedies of patriarchal simplicity, "spare diet and spring water," are all that are needed to guide the disorder to its natural cure. To this surest and safest of diuretics others must often be added, both to lessen dropsy and to avert the dangers of uræmia. The old rule is that, in recent cases, digitalis should be used; it seldom fails to increase the flow of urine, but I am not sure that it does not sometimes do so with some exasperation of the inflammatory action. The bitartrate and acetate of patash, which have a purgative as well as a diuretic action, may probably be safely resorted to; and in chronic cases as much as may be done harmlessly by diuretics may be accomplished by means of scoparium, nitre, and juniper. Cantharides and the more irritating agents of this class are generally distinctly injurious. Perhaps, next to a regulation of the diet, it is most important to secure a daily and somewhat loose action of the bowels. Purgatives lessen the vascular tension, which, in both acute and chronic cases, is a measure of their danger; and while it is not advisable too largely to divert the urinary fluids by severe catharsis, increased hardness of the pulse, and other more obvious aggravations of the general state, seldom fail to ensue upon constipation.

When cerebral uræmia is threatening, hard purging by elaterium or otherwise is essential. As a habitual laxative, a drug less used than it deserves to be—sulphate of potash—given two or three times a day in doses of from ten to twenty grains, is sometimes invaluable. It may be aided, if necessary, by Epsom salts or cream of tartar.—*Med. and Surg. Reporter.*

